# The Platelet Count in Cerebral Malaria, Is It Useful to the Clinician?

**DOI:** 10.4269/ajtmh.2010.09-0614

**Published:** 2010-07

**Authors:** Yamikani Chimalizeni, Kondwani Kawaza, Terrie Taylor, Malcolm Molyneux

**Affiliations:** Department of Paediatrics, College of Medicine, Blantyre, Malawi; Blantyre Malaria Project, Blantyre, Malawi; Malawi-Liverpool-Wellcome Trust Clinical Research Programme, College of Medicine, Blantyre, Malawi

## Abstract

We conducted this study to determine the prognostic significance of the platelet count in children with cerebral malaria. We studied children with cerebral malaria admitted to the pediatric research ward at Queen Elizabeth Central Hospital, Malawi. We analyzed 1,811 children with cerebral malaria and compared them with 521 children with bacterial meningitis. There was a significant difference in platelet counts between children with cerebral malaria and those with meningitis. Among children with cerebral malaria, there was no relationship between the platelet count and either the admission coma score or the eventual outcome. Those with malarial retinopathy were more thrombocytopenic than those without. Our results suggest that the platelet count is not prognostic in Malawian children with cerebral malaria.

## Introduction

Thrombocytopenia is common in all forms of malaria.^1^–^3^ Gerardin and others^4^ reported that in Senegalese children with severe malaria, those who died were more severely thrombocytopenic than those who recovered. In that study, children with severe malaria who had a platelet count of less than 100,000/μL (*N* = 110) were 6.3 (95% confidence interval [CI] 2–26) times more likely to die than those with a platelet count of above 100,000/μL. In a much larger study in coastal Kenya, Ladhani and colleagues^1^ reviewed 1,016 children admitted to a district hospital that fulfilled the World Health Organization (WHO) criteria for severe malaria, and found that the median platelet count was significantly lower among these children than in children admitted with mild, uncomplicated malaria. In the Kenya study, there was no association between the presence or severity of thrombocytopenia and a fatal outcome of the illness.

In an autopsy study in Blantyre, Malawi, 7 out of 31 children with an illness that fulfilled the WHO clinical definition of cerebral malaria proved at post mortem to have died of a cause other than malaria.^5^ In that study, the presence of specific retinal findings (“malarial retinopathy”) accurately predicted a malarial cause of death.^5^,^6^

Cerebral malaria is a common life-threatening complication of *Plasmodium falciparum* infection. Because the presence of retinopathy strengthens the diagnosis of this syndrome, we have examined the relationship between the circulating platelet count and the severity and outcome of cerebral malaria in Malawian children with and without malarial retinopathy.

## Materials and Methods

### Study area and population.

The study was conducted in children admitted to the pediatric research ward at the main referral hospital, Queen Elizabeth Central Hospital in Blantyre, Malawi, between 1995 and June 2007. For the purpose of this study, we retrospectively analyzed only children who were admitted with a Blantyre coma score of 2 or less, who had *P. falciparum* asexual parasites in the thick blood film, and in whom no explanation for coma other than malaria could be found. On admission, every child underwent detailed clinical assessment; from 1997 onward this included direct and indirect ophthalmoscopy. The age range of patients studied was 2 months to 171 months.

As a comparison group, we analyzed the platelet counts of children admitted during the same period with a diagnosis of bacterial meningitis. Bacterial meningitis was diagnosed on the basis of the following cerebrospinal fluid (CSF) findings: either a pathogenic bacterium seen on gram stain or cultured within 7 days; or a cell count greater than 100/μL containing a predominance of polymorphonuclear leukocytes.

### Laboratory procedures and other investigations.

Blood samples were collected in EDTA for immediate analysis by Coulter Counter. Thick blood films were stained with Fields stain and examined under oil-immersion. Asexual parasitemia was quantified by counting against white cells (thick film) and calculating density/μL from the total white cell count.

A lumbar puncture was done in all children except those in whom it was considered to be clinically dangerous to perform. Blood cultures were done in all patients and the blood glucose level was measured (repeatedly as indicated) in every case. Chest x-rays were done only when a special indication was present. In some patients with focal neurological signs a computed tomography (CT) scan was done when the facility was available, but this constituted a small minority of patients. Blood electrolytes were measured when judged clinically useful and when the facilities were available, but this too covered a minority of cases.

### Case management.

All children were started on intravenous quinine 20 mg/kg (loading dose) infused over 4 hours, and then 10 mg/kg 12 hourly until parasites cleared, followed by completion of antimalarial therapy by mouth. Anemic patients were transfused when necessary, and other supportive and antimicrobial therapy was given according to the guidelines of the Pediatric Department of the College of Medicine, University of Malawi.

### Statistical analysis.

Clinical data were analyzed using Stata 8.1 (Stata Corp., College Station, TX). The outcome of the illness was categorized as satisfactory (discharged well and with no neurological sequela) or unsatisfactory (died in hospital or had neurological sequela). Distributions were compared using the Mann Whitney test. Spearman's test was used to examine the correlation between parasite density and platelet counts. Proportions were compared by the Fisher's exact test. Thrombocytopenia was defined as a platelet count of less than 150,000/μL.

## Results

We analyzed 1,811 children who were admitted to the Pediatric Research Ward with the diagnosis of cerebral malaria between 1995 and 2007.

The age range of the children was 2 months to 14 years (median 34 months). Out of the 1,811 patients, 361 (19.9%) patients were admitted deeply unconscious with a Blantyre coma score of 0. Six hundred seventy-two (672) (37.1%) children were admitted with a Blantyre coma score of 1, and 778 (43.0%) had a coma score of 2.

The 1,317 (72.7%) patients made a full recovery, 188 (10.4%) had neurological sequel, and 306 (16.9%) died. The median admission parasite count was 104,160/μL.

### Bacterial meningitis and cerebral malaria compared.

We analyzed 521 children who were admitted with bacterial meningitis. The age range was 2 months to 168 months (median 13 months). Out of the 521 children, 82 patients had *P. falciparum* asexual parasitemia on the thick blood film; blood films were negative for parasites in the remaining 439. The median platelet count in patients with bacterial meningitis was 243,000/μL (245,000/μL in those who were parasitemic, and 215,000/μL in those who were parasitemic, *P* = 0.1036)

**Figure 1.**
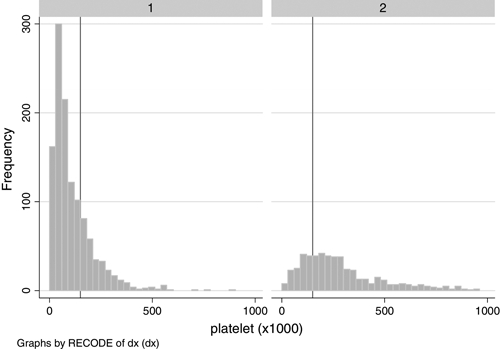
Distribution of platelets in two patient groups: (1) cerebral malaria (*N* = 1811) and (2) meningitis (*N* = 521).

In 1,811 patients admitted with a diagnosis of cerebral malaria, the median platelet count was 80,000/μL. There was a highly significant difference between the cerebral malaria and meningitis groups (Figure 1) (*P* < 0.0001).

### Relationship between the platelet count, coma score, and outcome.

There was no significant difference in the admission median platelet counts between those who were admitted with a Blantyre coma score of 0 and those admitted with a Blantyre coma score of either 1 or 2 (Table 1).

Among those admitted with a Blantyre coma score of 0, there was no significant difference in the median platelet count between those who had full recovery and those who either died or had neurological sequela (Table 1).

There was no significant difference in the outcome between patients who had an admission platelet count of less than 50,000/μL and those with a count of above 50,000/μL (Table 2). The outcomes were also similar when patients with an admission platelet count of less than 100,000/μL were compared with those having an admission platelet count of greater than 100,000/μL (Table 3). Similar analyses revealed no differences when restricted to patients with malarial retinopathy.

### Relationship between platelet count, presence of retinopathy, and outcome.

**Figure 2.**
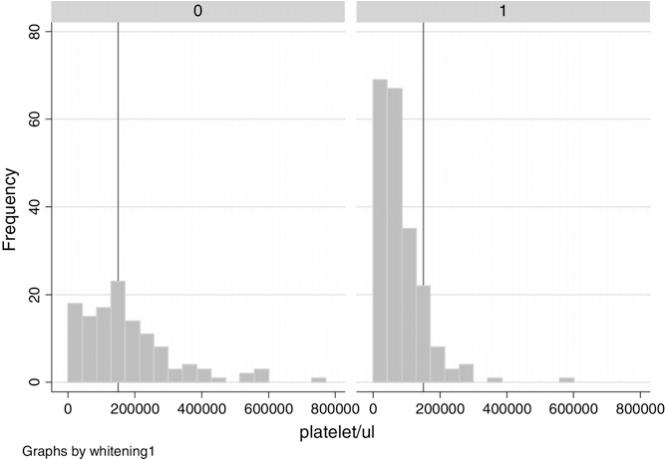
Distribution of admission platelet counts in two patient groups: (0) clinical cerebral malaria (CM) without retinopathy (*N* = 294) and (1) CM with retinopathy (*N* = 324) (*z* = 7.277, *P* = 0.0000).

There was a significant difference in the distribution of platelets between those children with clinical cerebral malaria who had retinopathy and those who did not (Figure 2). Children with cerebral malaria with retinopathy were twice as likely to be thrombocytopenic as were those without retinopathy.

Among patients who had cerebral malaria with retinopathy, there was no significant difference in the platelet counts between those who had full recovery and those who either died or had sequela (see Table 1).

The case fatality rate for patients who had cerebral malaria with retinopathy was 16.7%, whereas in the cerebral malaria group without retinopathy, it was 17.9%.

## Discussion

Thrombocytopenia has been shown to be present in all forms of malaria.^1^–^3^ Different mechanisms have been proposed to account for thrombocytopenia. Immune mediated mechanisms appear to be important, including immune destruction of circulating platelets,^7^ splenic pooling, and reduced platelet lifespan.^2^,^8^

In this study, we found a highly significant difference in the platelet counts between children who were admitted with a diagnosis of cerebral malaria and those with bacterial meningitis (*P* < 0.0001). The 18.7% of children in our study with bacterial meningitis had parasitemia, but the median platelet count in this group was not significantly lower than in children with meningitis who were not parasitemic. This suggests that in children admitted with acute bacterial meningitis, parasitemia may be an incidental finding not contributing to the child's disease.^9^,^10^

It has been suggested that platelets may play a role in the pathogenesis of cerebral malaria.^11^ Among children analyzed in this study, a subset of those who died were studied at autopsy and were found to have a significantly greater degree of platelet accumulation in brain microvasculature than children who died of severe malarial anemia or of non-malarial encephalopathies.^12^ It is not known whether this sequestration of platelets contributes to peripheral thrombocytopenia in malaria.

Few studies have been done to assess the clinical or prognostic significance of the circulating platelet count. In a study of 215 Senegalese children^4^ with severe malaria, those with a platelet count of less than 100,000/μL were more likely to die than those with a platelet count of greater than 100,000/μL (odds ratio [OR] = 6.31, 95% CI = 2.0–26.0). Among the 75 children with cerebral malaria in that study, those with a platelet count of less than 100,000/μL were similarly at greater risk of a fatal outcome than children with higher platelet counts (OR = 9.43, 95% CI = 2.3–54.4).

In this study of 1,811 prospectively enrolled children with cerebral malaria, we have shown no consistent relationship between the platelet count and outcome. We confirm previous reports that children admitted with a Blantyre coma score of 0 have a higher case fatality rate than children with less profound coma.^13^ Yet, we find no significant difference in the platelet counts between patient groups whether analyzed by their admitting coma score or by the outcome of their illness.

Children with malarial retinopathy, a physical sign strongly associated with intracerebral sequestration of parasites and suggestive of a diagnosis of “true” cerebral malaria,^5^,^6^ were twice as likely to be thrombocytopenic as those without retinopathy, and had significantly lower platelet counts (*P* < 0.0001). Even among children with retinopathy, however, there was no significant difference in the platelet counts between those that had full recovery and those that either died or had neurological sequela (*P* = 0.3).

We have shown that there was no difference in outcome between children who had a platelet count of less than 50,000 and those with a platelet count of greater than 50,000 (*P* = 0.097). The same analysis with a platelet count cutoff point of 100,000 revealed similar results (*P* = 0.36). This compares with the findings of a study in 2002 by Ladhani and others^1^ in which 1,369 children with malaria were analyzed (1,016 had severe malaria and 131 had cerebral malaria); no association between thrombocytopenia and outcome could be found.

In conclusion, we have confirmed the prominence of thrombocytopenia in malaria. Among children with cerebral malaria, those with retinopathy are more likely to be thrombocytopenic than those without retinopathy. However, we have found no relationship between the circulating platelet count and disease severity or outcome. The platelet count is not prognostic in Malawian children with cerebral malaria.

## Figures and Tables

**Table 1:** Distribution of clinical forms and outcome according to platelet counts in children with cerebral malaria in Blantyre, Malawi

Variables	Number	Platelet counts (×10^3^/mm^3^) median (IQ 25–75%) *P* value
Blantyre coma score (BCS) 0	237	90 (43–182) 0.06
1 or 2	943	78 (42–143)
Outcome (BCS = 0)	139	91 (42–187) 0.49
Full recovery died/sequela	96	83 (46–171)
Cerebral malaria with retinopathy	184	70 (41–115) 0.34
Full recovery died/sequela	58	71 (36–133)

**Table 2:** Platelet count and outcome in children admitted with cerebral malaria in Blantyre, Malawi

Platelet count/mm^3^	Full recovery	Died or sequela (%) *P* value
= < 50 000	327	124 (27.5%) 0.097
> 50 000	947	291 (23.5%)

**Table 3:** Comparison between platelet count and outcome in children admitted with cerebral malaria in Blantyre, Malawi

Platelet count/mm^3^	Full recovery	Died or sequel (%) *P* value
= < 100 000	698	216 (23.6%) 0.36
> 100 000	597	199 (25.6%)
